# Un textilome simulant une tumeur abdominale

**DOI:** 10.11604/pamj.2015.20.304.6588

**Published:** 2015-03-30

**Authors:** Ammar Mahmoudi, Faouzi Noomen

**Affiliations:** 1 Service de Chirurgie Générale et Digestive, CHU Fattouma Bourguiba de Monastir, Tunisie

**Keywords:** Chirurgie abdominale, iatrogénie, textilome, compresses, corps étrangers, abdominal surgery, iatrogenic, textiloma, gauze, foreign body

## Image en médecine

**Figure 1:**
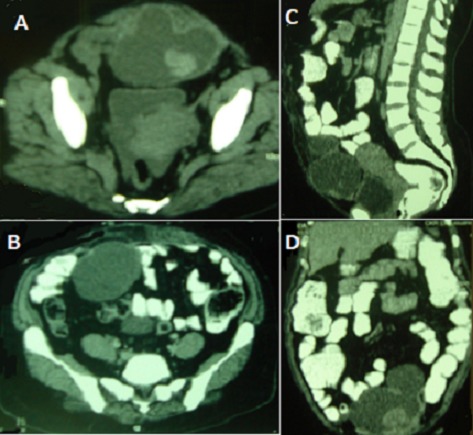
(A) scanner abdomino-pelvien en coupe axiale: masse pelvienne médiane pré-vésicale latéralisée à gauche de densité hétérogène avec une large composante liquidienne et une composante périphérique spontanément hyperdense; (B) scanner abdomino-pelvien en coupe axiale: masse pelvienne avec une large composante liquidienne. La masse prend corps avec les plans musculo-aponévrotiques; (C) scanner abdomino-pelvien en coupe sagittale: masse pelvienne médiane pré-vésicale de densité hétérogène avec une large composante liquidienne et une composante périphérique spontanément hyperdense. La masse prend corps avec les plans musculo-aponévrotiques; (D) scanner abdomino-pelvien en coupe frontale: masse pelvienne médiane de densité hétérogène avec une large composante liquidienne et une composante périphérique spontanément hyperdense. La masse prend corps avec les plans musculo-aponévrotiques

L'oubli d'un corps étranger textile est toujours possible au cours d'une chirurgie abdominale malgré les précautions draconiennes prises. La multiplicité et le manque de spécificité de ses présentations rendent son diagnostic difficile. Le meilleur traitement reste la prévention par le compte systématique des textiles et l'utilisation exclusive de compresses marquées, l'oubli d'une compresse étant toujours fautif. Nous rapportons le cas d'une patiente de 52 ans opérée il y a cinq ans pour une éventration ombilicale par la mise en place d'une prothèse qui présente une douleur abdominale à type de pesanteur évoluant depuis deux ans. A l'examen, on note une voussure sous-ombilicale de 15 x 10 cm. Le scanner abdomino-pelvien montre une masse pelvienne médiane pré-vésicale latéralisée à gauche mesurant 12 x 10 x 7,5 cm de densité hétérogène avec une large composante liquidienne et une composante périphérique spontanément hyperdense et quasiment non rehaussée par l'injection de produit de contraste. La masse prend corps avec les plans musculo-aponévrotiques notamment le muscle droit gauche évoquant une lésion tumorale musculaire primitive. Un kyste de l'ouraque dégénéré ou un hématome autours de la plaque décollée ont également été évoqués. La patiente a été opérée. Il existe une masse liquidienne intra-péritonéale de 15 cm de grand axe au contact du péritoine pariétal et reposant sur le dôme vésical. Il s'agit d'une cavité suppurée contenant la plaque qui est rétractée et un textilome (compresse). Il a été réalisé une évacuation du contenu de la cavité, une toilette et un drainage. Les suites opératoires étaient simples. Figure 1

